# Intra-Articular Polyacrylamide Hydrogel Injections Are Not Innocent

**DOI:** 10.1155/2014/150709

**Published:** 2014-08-13

**Authors:** Murat Tonbul, Mujdat Adas, Taner Bekmezci, Ahmet Duran Kara

**Affiliations:** ^1^Orthopedics and Traumatology Department, Private Reyap Hospital, Çorlu, 59860 Tekirdağ, Turkey; ^2^Orthopedics and Traumatology Department, Istanbul Okmeydani Hospital for Research and Education, Turkey; ^3^Orthopedics and Traumatology, Istanbul Liv Hospital, Turkey

## Abstract

Osteoarthritis is a chronic disorder characterized by joint cartilage degeneration with concomitant changes in the synovium and subchondral bone metabolism. Many conservative treatment modalities, one of which is intra-articular injections, have been described for the treatment of this disorder. Traditionally, hyaluranic acid and corticosteroids are the agents that have been used for this purpose. Recently, polyacrylamide hydrogels are being used widely. Biocompatibility, nonbioabsorbability, and anti-infectious effect obtained by silver addition made polyacrylamide hydrogels more popular. In this paper, we present a case and the method of our management, in whom host tissue reaction (foreign body granuloma, edema, inflammation, and redness induration) has been observed, as the first and unique adverse effect reported in the literature.

## 1. Introduction

Osteoarthritis is a chronic, progressive disorder characterized by joint cartilage degeneration associated with concomitant changes in the synovium and subchondral bone metabolism [1]. The most commonly affected joint is the knee, causing significant inabilities in 10% of patients older than 55 years [2]. The main and first treatment modality of osteoarthritis is conservative treatments including acetaminophen, nonsteroidal anti-inflammatory agents, exercise, and weight loss; intra-articular injections are gaining more popularity in recent years. In osteoarthritis, the decrease in intra-articular concentration and molecular weight of endogenous hyaluronic acid (HA) causes alterations in synovial fluid characteristics which in turn prompts cartilage degeneration and aggravations in symptoms [3].

Inra-articular HA injections have been recommended for osteoarthritis treatment [4, 5]. As an alternative, polyacrylamide hydrogels (PAH) have also gained popularity in recent years, especially in Asia [6, 7]. However, it is a new treatment option; it should be critically investigated before treating our patients.

Polyacrylamide hydrogels are biostable, high grade biocompatible, synthetic polymers including silver ions that are used as filling materials. They contain 4.5 ± 1.5% polyacrylamide, 95.5 ± 1.5% distillated water, and 0.01-0.02% silver ions [8, 9]. In particular after esthetic surgeries, transient surgical side pain, hematoma, irregularity, gel deposition, and asymmetry, tissue reactions including infection, foreign body granuloma, edema, inflammation, tenderness, and sensitivity, and adverse effects including gel migration and induration were very rarely reported [10].

In this case report we aim to present a case, in which relevant adverse effects followed the intra-articular treatment of knee osteoarthritis in a patient.

## 2. Case

**Figure 1:**
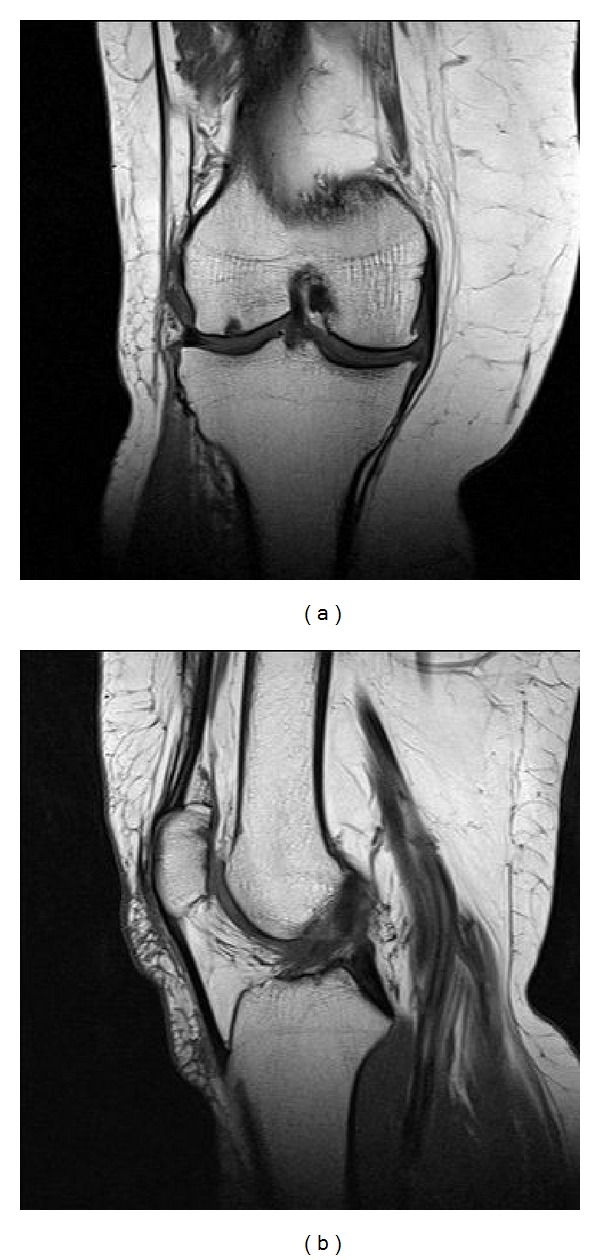
Preoperative T1-weighted MR image of the right knee, revealing complete tear of the medial meniscus, degeneration of the tibiofemoral joint cartilage, and chondromalacia of the patella.

A 64-year-old female patient was admitted to our outpatient clinic with the complaint of bilateral knee pain being more significant on right side. She complained of decrease of the walking distance and rarely night pain. She did not use any canes or walker before. Her symptoms were present for about 5 years. She stated that the previous medical treatment and physical rehabilitation procedures did not have any effect. Magnetic resonance imaging of her right knee revealed a complete tear on medial meniscus, degeneration on tibiofemoral joint cartilage, and chondromalacia of the patella (Figure 1). Via the arthroscopic intervention, partial medial meniscectomy and cartilage debridement because of the outerbridge grade 2-3 chondropathy in the medial femoral condyle have been performed to the patient.

**Figure 2:**
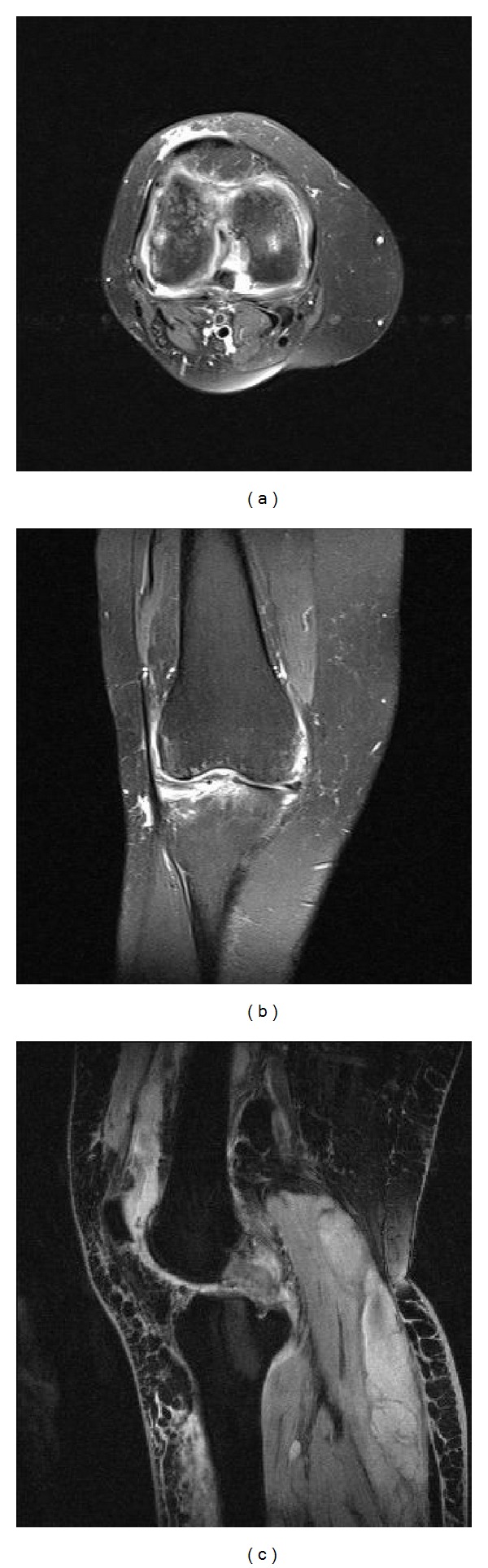
Postoperative T2-weighted MR images of the right knee, revealing effusion in the knee joint and popliteal fluid collection.

**Figure 3:**
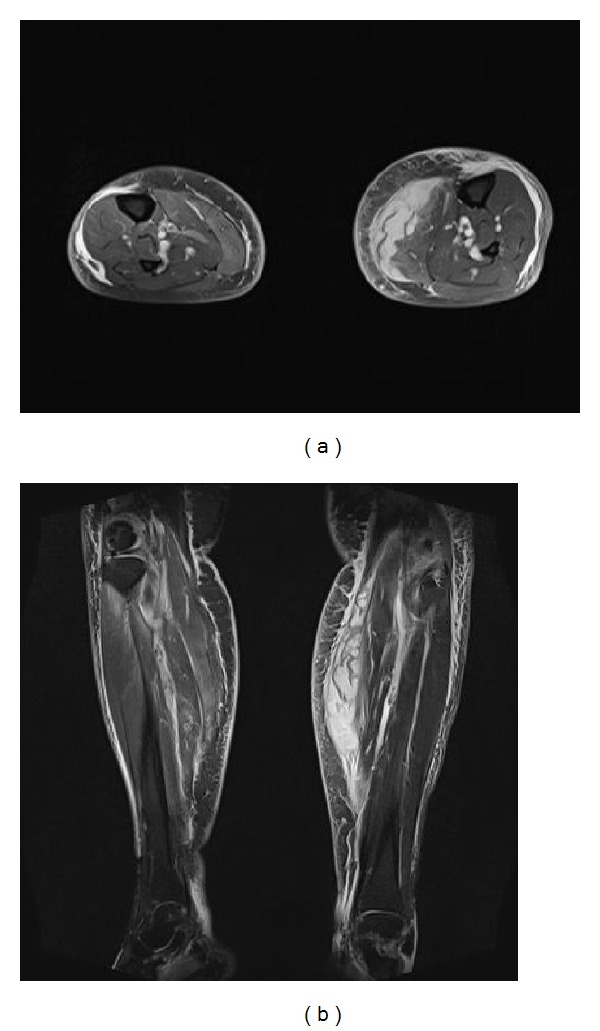
Postoperative T1-weighted MR images of both crures, revealing fluid collection in the posterior compartment.

Immediately after the operation, 2.5 mL polyacrylamide hydrogel (Noltrex, Bioform, Moscow, Russia) was injected with standard approach to the knees in order to diminish the cartilage damage. After injection, in first 24 hours, classical inflammatory responses, tumor, dolor, rubor, calor, and functio laesa, were observed in the right knee. This condition was considered as a synovial reaction and nonsteroidal anti-inflammatory agents and intermittent local cold administrations were applied. There were no reactions on left side. Since the right knee got worse, joint aspiration was performed and a semisolid, semitransparent, scentless, yellow colored material was obtained. As this material was the Noltrex itself, no histologic, immunologic, or chemical analysis has been performed. Moreover, in microbiological evaluation there were no bacteria isolated; in gram staining there was considerable amount of leucocytes in some areas but there were no gram (+) or (−) bacteria. Later, in the 5th day after the injection, because her complaints increased, after an MR imaging, arthroscopic joint debridement was performed (Figure 2). The aforementioned scentless, semisolid and semitransparent, yellow-colored material was completely drained from the joint. There were widespread synovial hypertrophy and patchy hemorrhagic spots inside the joint. No bacteria were isolated from the material obtained from arthroscopic debridement. Two days later from the second operation, there were swelling and pain in the whole extremity especially on the popliteal fossa. Lower extremity venous Doppler ultrasound performed with the prediagnosis of deep vein thrombosis, a collection focused on Baker cyst with a semisolid echogenity in the popliteal region was determined. The collection was drained with a catheter placed with ultrasonic guidance. Five days later, the swelling decreased and the catheter was withdrawn. The results of joint aspiration performed to the left knee, to which only intra-articular injection was performed concomitantly, due to the swelling and tenderness were similar to that of the right knee. Because of the increased complaints of her left knee, an arthroscopic debridement via the anterolateral portal was also performed at the ninth day. No bacteria were isolated in the microbiological evaluation. There were widespread synovial hypertrophy and patchy hemorrhagic spots inside the joint. Similar to right knee, there was also a collection on popliteal region of left knee. Twelve hours later, it has been determined that the Baker cyst was ruptured and the collection was spread through the posterior crural muscles. Because of its septal and multifocal structure, it could not be catheterized (Figure 3). After 24 hours of follow-up with elevation and cold compress, aspirative drainage was performed to the fluctuating region on back side of cruris, and serohemorrhagic fluid was drained. At the end of the sixth day the drain was withdrawn as the swelling decreases. The patient was able to walk with a slight limping and she had to use a walker about 3 months later of the first injection.

## 3. Discussion

Polyacrylamide hydrogels are biostable and very commonly used as filling materials especially in esthetic and urologic surgeries [10, 11]. Their popularity is increasing, particularly in the practice of orthopedics recently. Zar et al., in their study of 527 patients, have reported an adverse effect profile after injection as inflammable pain in 6.8% of patients on injection area, joint pain in 9.3% of patients, and joint effusion in 0.05% of patients [7]. All these adverse effects were resolved with local cold compress and acetaminophen treatments in 3 days. In none of the patients, joint aspiration or debridement was required.

To our best knowledge, the case we presented in this paper is unique in the literature highlighting the immediate adverse effect of the polyacrylamide hydrogel. Since it occurred both in the operated and in the nonoperated sides we could not explain it with only operation induced inflammatory response increased by injection such a “foreign body.” Moreover, since there were not any bacteria isolated in direct evaluations or cultures, it has been thought as an inflammatory procedure. Early surgical interventions performed in order to drain out and debride the hydrogel and conservative treatment modalities of both basic orthopedic disciplines and manufacturer's guidelines were absolutely achieved.

During the secondary operative interventions, we had had a consensus on that, before treating patients with such new methods, critical investigations have to be made. Probably in this patient we have altered the course of her osteoarthritis, by biologically causing intra- and extra-articular fibrosis. This may also have altered the possible treatments, such as arthroplasties and their postoperative rehabilitations.

In conclusion, the treatment of adverse effects of PAH is not as easy as known or thought. The correct diagnosis of complication is essential in order to choose the appropriate treatment and abstain from unnecessary surgical interventions. However, since polyacrylamide hydrogels are biostable, in case of any suspicion, mechanical cleaning should be performed immediately to relief the patient.
